# Effect of age at onset on cortical thickness and cognition in posterior cortical atrophy

**DOI:** 10.1016/j.neurobiolaging.2016.04.012

**Published:** 2016-04-25

**Authors:** Aida Suárez-González, Manja Lehmann, Timothy J. Shakespeare, Keir X.X. Yong, Ross W. Paterson, Catherine F. Slattery, Alexander J.M. Foulkes, Gil D. Rabinovici, Eulogio Gil-Néciga, Florinda Roldán-Lora, Jonathan M. Schott, Nick C. Fox, Sebastian J. Crutch

**Affiliations:** a Dementia Research Centre, Department of Neurodegenerative Disease, UCL Institute of Neurology, University College London, London, UK; b Department of Neurology, University Hospital Virgen del Rocio, Seville, Spain; c Memory and Aging Center, University of California San Francisco, San Francisco, California, USA; d Department of Radiology, University Hospital Virgen del Rocio, Seville, Spain

**Keywords:** Posterior cortical atrophy, Age at onset, Neuroimaging, Cortical thickness, Atypical Alzheimer disease

## Abstract

Age at onset (AAO) has been shown to influence the phenotype of Alzheimer’s disease (AD), but how it affects atypical presentations of AD remains unknown. Posterior cortical atrophy (PCA) is the most common form of atypical AD. In this study, we aimed to investigate the effect of AAO on cortical thickness and cognitive function in 98 PCA patients. We used Freesurfer (v5.3.0) to compare cortical thickness with AAO both as a continuous variable, and by dichotomizing the groups based on median age (58 years). In both the continuous and dichotomized analyses, we found a pattern suggestive of thinner cortex in precuneus and parietal areas in earlier-onset PCA, and lower cortical thickness in anterior cingulate and prefrontal cortex in later-onset PCA. These cortical thickness differences between PCA subgroups were consistent with earlier-onset PCA patients performing worse on cognitive tests involving parietal functions. Our results provide a suggestion that AAO may not only affect the clinico-anatomical characteristics in AD but may also affect atrophy patterns and cognition within atypical AD phenotypes.

## 1 Introduction

Alzheimer’s disease (AD) is a neurodegenerative disease that typically presents with insidious and progressive memory loss and early atrophy in the hippocampus and medial temporal lobes (McKhann et al., 2011). Histopathologically, AD is characterized by the deposition of β-amyloid neuritic plaques, and intraneuronal neurofibrillary tangles, which typically first appear in entorhinal, limbic, and then neocortical regions and which are associated topographically with neuronal loss (Braak and Braak, 1991; Gomez Isla et al., 1997). The age at onset (AAO) of the disease has increasingly been recognized as a factor that may influence both the pattern of atrophy and the clinical symptoms in patients with AD (Kaiser et al., 2012). Early-onset AD, traditionally defined as onset before 65 years, differs from late-onset AD in that the pattern of cerebral atrophy in these younger individuals is more widespread, less prominent in medial temporal regions and more severe in posterior cingulate, temporo-parietal areas, and precuneus (Frisoni et al., 2005, 2007). Early-onset AD patients also show greater cortical atrophy and hypometabolism than late-onset AD at the same disease clinical stage (Kim et al., 2005). From a pathological point of view, studies have reported larger burden of pathology in younger patients (Marshall et al., 2007) and more widespread and more pronounced burden outside the medial temporal lobe compared with patients with late-onset AD. Atypical variants of AD are also commonly associated with an early AAO (Greicius et al., 2002) and more often present with nonmemory symptoms including visuospatial function, apraxia, and language deficits (Galton et al., 2000; Schott and Warren, 2012; Van der Flier et al., 2011).

Posterior cortical atrophy (PCA) is the most common atypical form of AD. It is characterized by insidious and gradual visual complaints in the absence of primary ocular disease (see Crutch et al., 2012 for a review). Symptoms typically progress over time to include other functions such as praxis and calculation and memory, whereas insight is relatively preserved until later stages (Tang-Wai et al., 2004). PCA patients show atrophy mainly in parieto-occipital regions (Lehmann et al., 2011) with atrophy patterns progressing to a more global pattern with advancing disease stage (Lehmann et al., 2012). Pathologically, patients with PCA show fairly similar appearances to typical AD, although there are differences in the distribution of the pathological changes, with greater density of neurofibrillary tangles described in the parietal and occipito-temporal junction, and lower amyloid burden in the hippocampus (Hof et al., 1997; Tang-Wai et al., 2004). Although PCA is typically considered a young-onset form of AD, it can also occur in older patients. However, the pathophysiological underpinning of different ages at onset remains unknown.

In this study, we aimed to investigate the effect of AAO on cortical thickness in PCA. As early AAO in AD has been linked to a higher proportion of focal phenotypes, we expected individuals with earlier-onset PCA to show a more focal presentation compared with those with later-onset PCA. We therefore hypothesized that PCA patients with earlier AAO would have greater loss in cortical thickness in posterior regions compared with later-onset PCA.

## 2 Methods

### 2.1 Participants

This study involved a total of 98 PCA patients. Patients were recruited at 3 specialist centers: 81 patients at the Dementia Research Centre (DRC) at the National Hospital for Neurology and Neurosurgery London (UK), 9 patients at the University Hospital Virgen del Rocio (HUVR) Memory Disorders Unit (Spain), and 8 patients at University of California San Francisco (UCSF) Memory and Aging Center (US). Informed consent was obtained from all subjects and the study had local ethics committee approval. All patients met clinical diagnostic criteria for PCA (Mendez et al., 2002; Tang-Wai et al., 2004), fulfilled criteria for probable AD (Dubois et al., 2014; McKhann et al., 1984, 2011), and had to have a suitable MRI scan available. Patients at UCSF and HUVR were only included if they had undergone the same neuropsychological battery as patients from the DRC. For all centers, patients underwent comprehensive neurological examination. AAO was ascertained by asking participants or their caregivers when they first experienced symptoms. A group of 91 control subjects was included for comparison of the imaging data, matched for gender, age, scanner field strength, and site (64 DRC, 19 HUVR, and 8 UCSF). Demographics and clinical data are shown in Table 1.

### 2.2 Background neuropsychological testing

Detailed neuropsychological assessment was available for 68 of the 98 participants (52 DRC, 9 HUVR, 7 UCSF) and consisted of the mini-mental state examination (MMSE; Folstein et al., 1975), digit span forward and backward, short Recognition Memory Test (Warrington, 1996), graded difficulty arithmetic test (Jackson and Warrington, 1986), graded difficulty spelling test (Baxter and Warrington, 1994), assessment of apraxia through gesture production, and the subtest of figure-ground discrimination, fragmented letters, object decision, dot counting, and number location from the Visual Object And Space Perception Battery (Warrington and James, 1991).

### 2.3 Image acquisition and processing

T1-weighted volumetric MR scans were acquired on 5 different scanners (two 3T Trio (DRC and UCSF), 1.5T Intera (HUVR), and two 1.5 Signa units [DRC]) using spoiled gradient recalled or gradient echo (MPRAGE) sequences. The scans consisted of full brain coverage coronal or sagittal slices running between 124 and 208 contiguous slices of 1.5 or 1.0 mm. Full details of imaging parameters are shown in the Supplementary Methods, and site and scanner distribution in earlier and later PCA are shown in Supplementary Table 1. For patients with neuropsychological assessment, all scans were performed within 6 months from cognitive testing. All scans were transferred to a Linux workstation for analysis.

Cortical thickness measurements were made using the freely available software Freesurfer, version 5.3.0 (http://surfer.nmr.mgh.harvard.edu/). The detailed procedure for the surface construction has been described and validated in previous publications (Dale et al., 1999; Fischl and Dale, 2000). Briefly, the image processing included intensity normalization, removal of nonbrain tissue, segmentation, surface inflation, and topological correction. Cortical thickness was then calculated as the closest distance from the grey/white boundary to the grey/CSF boundary at each vertex on the surface. Cortical thickness was smoothed with a 20-mm full-width at half height Gaussian kernel to reduce local variations in the measurements for further analysis. Surfaces were checked and manual edits were performed in cases of gross inaccuracies using the Freesurfer editing tools. Values for estimated total intracranial volume were also obtained from Freesurfer.

### 2.4 Statistical analysis

Both neuropsychological and neuroimaging data were analyzed using 2 different approaches: (1) comparing earlier- and later-onset PCA by splitting the PCA sample using the median AAO = 58 years as the cut-off value, and (2) using AAO as a continuous variable within the whole PCA sample.

#### 2.4.1 Neuropsychological testing

The normality of score distribution was investigated for each neuropsychological test using the Kolmogorov–Smirnov test, and differences between groups were calculated using unpaired *t* test or *U* Mann–Whitney where scores were not normally distributed. Correlation between cognitive scores and AAO was performed using Spearman’s correlation coefficient. Additional analyses were conducted correcting for MMSE and disease duration (in 2 separate models).

#### 2.4.2 Cortical thickness

##### 2.4.2.1 Controls versus PCA and earlier- versus later-onset PCA

Regional cortical thickness variations between controls and PCA, and earlier- and later-onset PCA were assessed using a vertex-by-vertex general linear model performed with the Surfstat software for Matlab (http://www.stat.uchicago.edu/~worsley/surfstat/). Cortical thickness was modeled as a function of group (controls, earlier-onset PCA, later-onset PCA), controlling for age (mean centered), gender, total intracranial volume, site, and field strength. Group differences between controls and PCA subgroups were corrected for multiple comparisons (family-wise error [FWE], *p* < 0.05), whereas differences between PCA subgroups (earlier- vs. later-onset PCA), which did not survive multiple comparison correction, are shown at an uncorrected statistical threshold of *p* < 0.05 and as percent difference maps.

##### 2.4.2.2 AAO as a continuous variable

Associations between AAO and cortical thickness were further explored using AAO as a continuous variable using the same covariates as in the group comparisons. Results are shown as statistical p-maps (uncorrected at *p* < 0.05) and as effect size maps showing correlation coefficients. The analysis was repeated including MMSE and disease duration (in 2 separate models) to adjust for the effects of disease severity.

## 3 Results

### 3.1 Neuropsychological testing

In the subset of patients with neuropsychological data available earlier- and later-onset PCA patients differed in age and AAO (by definition) but were matched for disease duration and MMSE (Table 2). Comparing cognitive scores between earlier- and later-onset PCA, we found that earlier-onset PCA was associated with statistically significantly worse performance on digit span forward and backward (*p* = 0.034 and *p* = 0.029 respectively), and a trend toward worse performance in calculation and spelling (*p* = 0.05)—all of them tasks associated with dominant-parietal function. However, there were no differences in memory, praxis, or visual tests. Using AAO as a continuous variable, a positive correlation was found with performance on digit span forward (R^2^ = 0.53, *p* = 0.001), calculation (R^2^ = 0.48, *p* = 0.013) and spelling (R^2^ = 0.29, *p* = 0.026). Similar results were found when correcting for MMSE, whereas the correlation between AAO and spelling became nonsignificant after correcting for disease duration (*p* = 0.078).

### 3.2 Cortical thickness

**Fig. 1:**
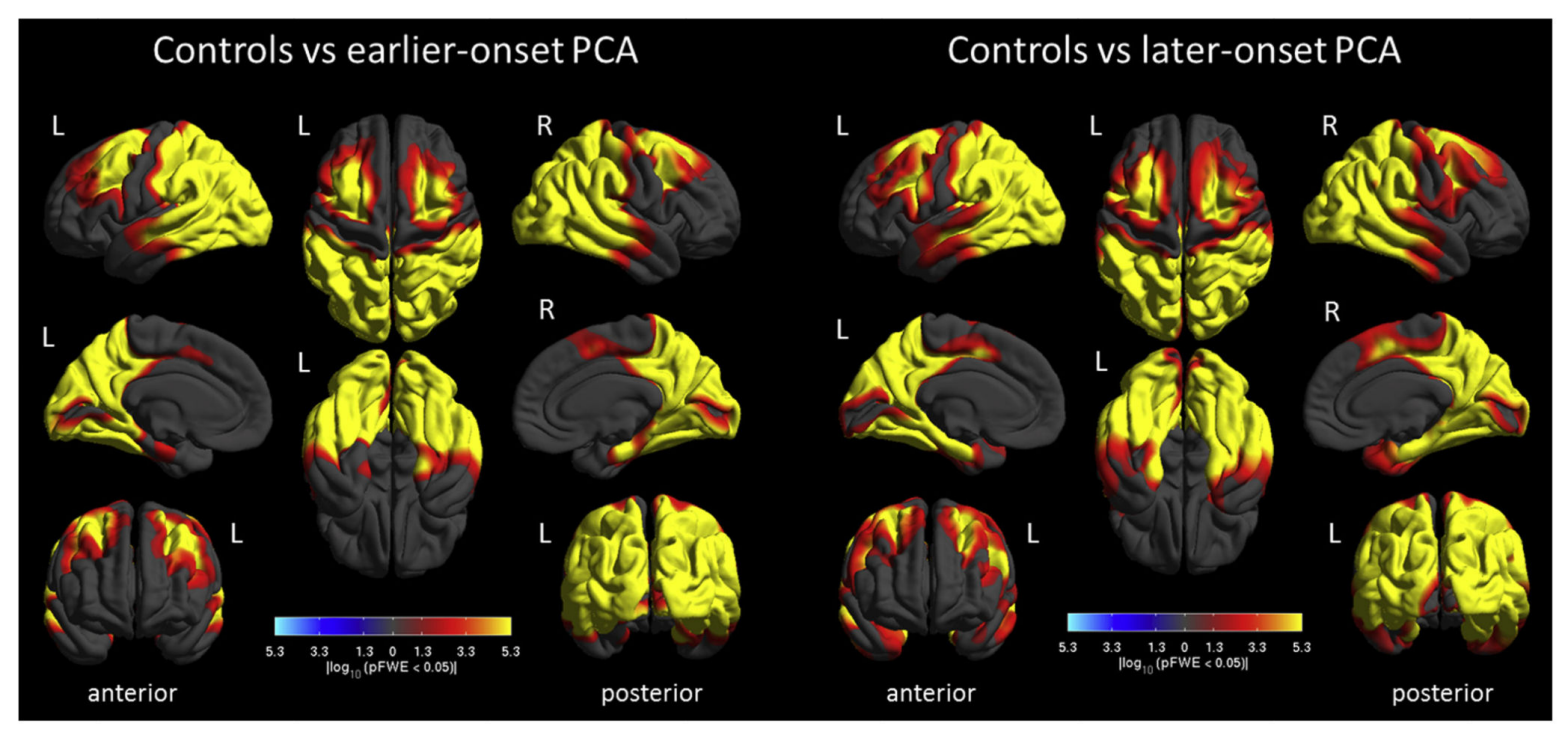
Patterns of cortical thickness in earlier-onset PCA (left) and later-onset PCA (right) compared with controls. Shown are statistical p-maps corrected for multiple comparisons (FWE *p* < 0.05). Warmer colors (yellow and red) indicate lower cortical thickness in the PCA patients, whereas cooler colors (blue) represent lower cortical thickness in controls (which did not yield any significant results). Significant cortical thinning, relative to the control groups, is seen in widespread posterior regions for both the earlier- and later-onset PCA groups. Abbreviations: FWE, family-wise error; PCA, posterior cortical atrophy.

**Fig. 2:**
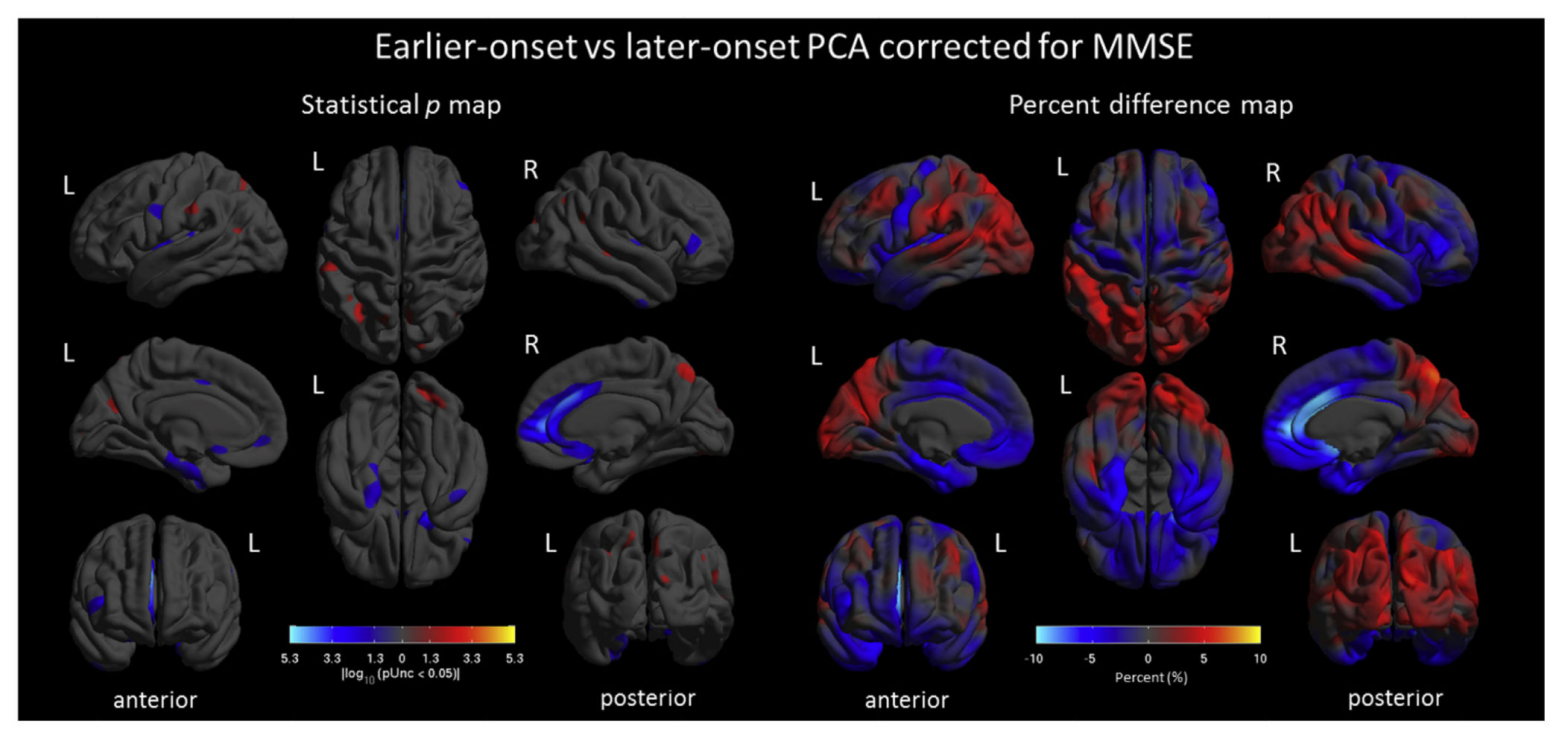
Patterns of cortical thickness in earlier- versus later-onset PCA corrected by MMSE. Shown are statistical p-maps (left) uncorrected for multiple comparisons (*p* < 0.05) and percent difference maps (right). Warmer colors (yellow and red) indicate lower cortical thickness in earlier-onset PCA compared with later-onset PCA, whereas cooler colors (blue) represent the reverse contrast. The percentage difference maps show that earlier-onset PCA has greater posterior and lateral cortical thinning, whereas later-onset PCA show greater anterior cortical thinning. Abbreviations: MMSE, mini-mental state examination; PCA, posterior cortical atrophy.

Fig. 1 shows differences in cortical thickness between controls and PCA subgroups (percentage maps are shown in Supplementary Fig. 1). Both earlier- and later-onset PCA groups showed significantly lower cortical thickness in occipital, parietal, temporal posterior, and motor cortices compared with controls, whereas prefrontal cortices remained relatively preserved (*p* < 0.05, FWE corrected). The direct comparison between earlier- and later-onset PCA corrected by MMSE (Fig. 2) showed lower cortical thickness in posterior regions in earlier-onset PCA, including the left angular gyrus, precuneus, and parieto-occipital sulcus, whereas later-onset PCA showed lower thickness in right anterior cingulate gyrus and prefrontal areas (*p* < 0.05, uncorrected).

**Fig. 3:**
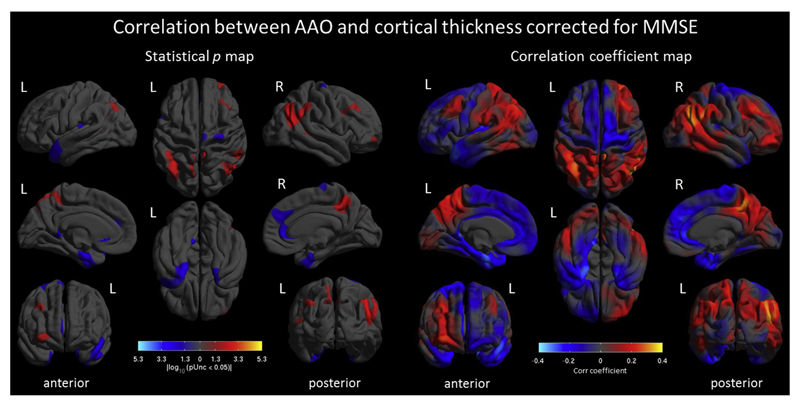
Correlation between AAO and cortical thickness corrected by MMSE. Shown are statistical p-maps (left) uncorrected for multiple comparisons (*p* < 0.05), and correlation coefficient maps (right). Warmer colors indicate positive correlations, that is, earlier AAO is associated with lower cortical thickness, whereas cooler colors show negative correlations where later AAO is associated with lower thickness. Abbreviations: AAO, age at onset; MMSE, mini-mental state examination.

Using AAO as a continuous variable (corrected by MMSE) produced similar results (Fig. 3), with earlier AAO being associated with lower cortical thickness in the right temporo-parieto-occipital junction as well as left inferior parietal lobe, bilateral precuneus, and a small region in the right middle frontal gyrus (*p* < 0.05, uncorrected). In contrast, later AAO was associated with lower cortical thickness in anterior cingulate cortex, prefrontal lobe, and temporal poles (*p* < 0.05, uncorrected). Excluding MMSE and including disease duration as covariate in different models produced similar results (Supplementary Figs. 2, 3, 4 and 5).

## 4 Discussion

This is the first study to report the effects of AAO on cognition and cortical thickness in a large sample of patients with PCA. We found that PCA patients with an earlier AAO showed lower cortical thickness in parietal areas, whereas later-onset PCA patients showed greater involvement of anterior regions. Atrophy patterns were consistent with cognitive data which showed that earlier AAO was associated with greater deficits in parietal lobe functions (digit span and calculation). Consistent with our initial hypothesis, we found a greater involvement of parietal regions in PCA patients with earlier AAO. Interestingly, we did not find evidence of an association between AAO and cortical thickness of the occipital lobe.

Previous studies have validated the use of cortical thickness as a method to quantify and localize cortical thinning in PCA and demonstrated lower thickness in occipital and parietal lobe in PCA compared with controls (Lehmann et al., 2011), which is in accordance with our results. Furthermore, in this study, there are suggestions that the distribution of cortical thickness in PCA varied according to AAO. Although not significant when correcting for multiple comparisons, greater thinning in precuneus and parieto-occipital cortex was found in earlier-onset individuals, whereas later-onset PCA patients showed greater involvement of prefrontal and anterior cingulate cortices. This suggests that AAO not only has an effect on the severity of parietal atrophy but also on the distribution of cortical thinning. Interestingly, this pattern of involvement found in the later-onset group is, although beyond our initial hypothesis, in keeping with our initial theory, this is, patients with later-onset PCA showing a pattern of cortical thinning less restricted to posterior areas. Unfortunately, because our cognitive battery only included a limited number of tests assessing prefrontal functions, it was not possible to assess the neuropsychological effects of this finding in the present study.

Greater thinning of the parietal cortex along with poorer performance on cognitive tasks related to parietal function showed a tendency to be associated with an earlier onset of PCA. This may suggest that this area holds a greater pathological burden in this subgroup. Tau is closely linked to hypometabolism and clinical symptoms in AD (Ossenkoppele et al., 2015), and previous studies have shown greater burden of both amyloid and tau pathology in early-onset AD (Bigio et al., 2002; Marshall et al., 2007). It has been suggested that this might be due to the fact that greater cognitive reserve in younger individuals requires more pathological change (i.e., a more advanced pathological stage) for them to exhibit the same degree of cognitive deterioration than their older counterparts (Marshall et al., 2007).

The factors and mechanisms causing some patients to develop the disease earlier in life are not well understood, although AAO might at least be partially associated with genetic risk factors. For instance, earlier AAO has been associated with the presence of at least 1 ApoE ε4 allele in AD, and homozygous ε4 allele carriers develop AD up to 10 years earlier than individuals who do not carry this allele (Blacker et al., 1997). It is possible that people with earlier-onset PCA may possess genetic risk factors that predispose them to develop both earlier AD and PCA and may potentially lead to the specific clinico-anatomical phenotype of this group. Unfortunately, ApoE status was not available for the present study.

In our study, there was an association between earlier AAO and significantly worse performance in 4 dominant-parietal tasks (digit forward and backward, calculation, and spelling), suggesting a greater involvement of parietal areas consistent with neuroimaging findings. Forward and backward digit span are tasks widely used to assess short-term and working memory. An important component of working memory is the phonological store, which is essential for auditory information to be held for a few seconds and is underpinned by the inferior parietal cortex. In parallel to the phonological store, the visuospatial sketchpad (supported by parietal cortices) serves the processing of visual and spatial information and has been shown also to be crucially involved in backwards digit span performance (Li and Lewandowsky, 1993, 1995). We also found a significant association between worse calculation skills and earlier AAO which is consistent with the greater parietal thinning in the earlier-onset subgroup because calculation performance is underpinned by the left dorsal angular gyrus and medial parietal cortex (Gruber et al., 2001),. Regarding the deficits in spelling, these have been related to atrophy in the supramarginal and posterior-inferior temporal/fusiform gyrus (Philipose et al., 2007) and specifically in AD spelling is associated with a left-lateralized cortical network involving the posteriorinferior temporal lobe and superior parietal cortex (Rodriguez-Ferreiro et al., 2014). Finally, it might have been expected that in the subsample of 68 PCA (with neuropsychological assessment available) the earlier-onset patients showed lower MMSE because of their greater parietal impairment. Contrary to this, performance in MMSE was similar in both groups. This might be due to the fact that the MMSE is an imperfect measure of disease severity and that test of digit span, graded difficulty arithmetic calculation, and graded difficulty spelling (main indicators of the worse parietal dysfunction in the earlier-onset group) are more sensitive and specific to parietalmediated functions than the MMSE.

This study has a number of limitations that should be taken into account. Neither autopsy-confirmed diagnosis nor pathophysiological biomarkers were available in the present study and therefore, it is possible that non-AD cases were included in the current sample. Owing to the multicentre nature of our study, scans were acquired at different scanners and sites. We therefore included site and field strength as covariates in our analyses.

In summary, our data provide preliminary evidence that AAO might affect the clinico-anatomical phenotype in PCA. Individuals with earlier AAO may have a greater degree of cortical thickness loss in the parietal cortex and greater cognitive deficits related to this region. In contrast, patients with later AAO may have a greater degree of cortical thickness loss in prefrontal and anterior cingulate cortices compared with earlier-onset PCA. These atrophy patterns may suggest that the earlier the AAO the more severe the parietal involvement and the more restricted the thickness loss to posterior structures. Our findings extend previous research on the effects of AAO on the clinico-anatomical manifestation of AD and may help to unravel the expression of selective vulnerabilities in the AD spectrum.

## Supplementary Material

**Appendix A. Supplementary data**

Supplementary data associated with this article can be found, in the online version, at http://dx.doi.org/10.1016/j.neurobiolaging.2016.04.012.

Supplementary Figure 1

Supplementary Figure 2

Supplementary Figure 3

Supplementary Figure 4

Supplementary Figure 5

Supplementary Methods

Supplementary Table 1

## Figures and Tables

**Table 1:** Demographics of the control group, total sample of PCA, and earlier- and later-onset PCA subgroups

	Controls	PCA	PCA ≤ 58	PCA > 58	*p*
N	91	98	49	49	
Gender	33 m/58f	40 m/58f	14 m/35f	26 m/23f	–
Age	64 ± 5	64 ± 7	58 ± 4	68 ± 5	<0.0001
Age at onset	–	59 ± 7	53 ± 3	63 ± 5	<0.0001
MMSE	–	19 ± 2	18 ± 5	21 ± 5	0.04
Disease duration, y	–	4.8 ± 0	5 ± 2	5 ± 2	0.77
Scanner (3T, 1.5T)	51, 40	53, 45	27, 22	26, 23	–

Data shown as mean (standard deviation). Key: MMSE, mini-mental state examination; PCA, posterior cortical atrophy.

**Table 2:** Demographics and neuropsychological data of the subgroup of 68 PCA patients

	PCA	PCA ≤ 58	PCA > 58	*P*
N	68	34	34	
Age	63 ± 7 (49–85)	58 ± 4	69 ± 5	**<0.0001**^c^
Age at onset	59 ± 7 (44–82)	53 ± 4	64 ± 5	**<0.0001**^c^
Disease duration, y	4 ± 2 (0–11)	4 ± 2	4 ± 2	0.77^c^
MMSE	19 ± 5 (8–29)	19 ± 5	20 ± 5	0.56^b^
Neuropsychology (n/N)^a^				
Digit span forward	5 ± 1 (3–9)	5 ± 1 (3–8)	6 ± 1.4 (4–9)	**0.034**^c^
Digit span backward	2 ± 0 (0–5)	2 ± 0 (0–4)	3 ± (2–5)	**0.029**^c^
Short RMT (words) (60/68)	19 ± 4 (7–25)	19 ± 4 (7–25)	19 ± 4 (9–25)	0.89^c^
Short RMT (faces) (28/68)	18 ± 4 (10–25)	18 ± 5 (10–25)	18 ± 3 (13–250)	0.86^b^
Calculation (68/68)	10 ± 5 (0–21)	8 ± 5 (0–19)	11 ± 5 (0–21)	**0.05**^c^
Spelling (57/68)	11 ± 6 (0–20)	9 ± 6 (0–20)	12 ± 6 (1–20)	**0.05**^b^
Gesture production (50/68)	12 ± 3 (3–15)	12 ± 3 (3–15)	12 ± 2 (5–15)	0.59^c^
VOSP				
Figure ground (67/68)	16 ± 3 (0–20)	16 ± 2 (10–20)	15 ± 4 (0–20)	0.74^c^
Fragmented letters (67/68)	6 ± 6 (0–19)	6 ± 6 (0–19)	3 ± 4 (0–19)	0.07^c^
Object decision (68/68)	10 ± 5 (0–20)	10 ± 4 (4–20)	9 ± 4 (0–18)	0.46^c^
Dot counting (67/68)	5 ± 3 (0–10)	4 ± 3 (0–10)	4 ± 3 (0–10)	0.75^c^
Number location (65/68)	3 ± 3 (0–10)	3 ± 3 (0–10)	2 ± 3 (0–9)	0.85^c^

Results expressed in mean (standard deviation) and range. Bold means statistically significant or trend toward statistically significant differences. Key: MMSE, mini-mental state examination; PCA, posterior cortical atrophy; RMT, Recognition Memory Test; VOSP, Visual Object and Space Perception Battery.

a Number of individuals that completed the test.

b *t*-test.

c *U* Mann–Whitney.
