# Supplementary figures and images for: Effect of age at onset on cortical thickness and cognition in posterior cortical atrophy
Source: Neurobiol Aging. Author manuscript; Available in PMC 2016 Aug 1. (PMC4926954; doi:10.1016/j.neurobiolaging.2016.04.012)

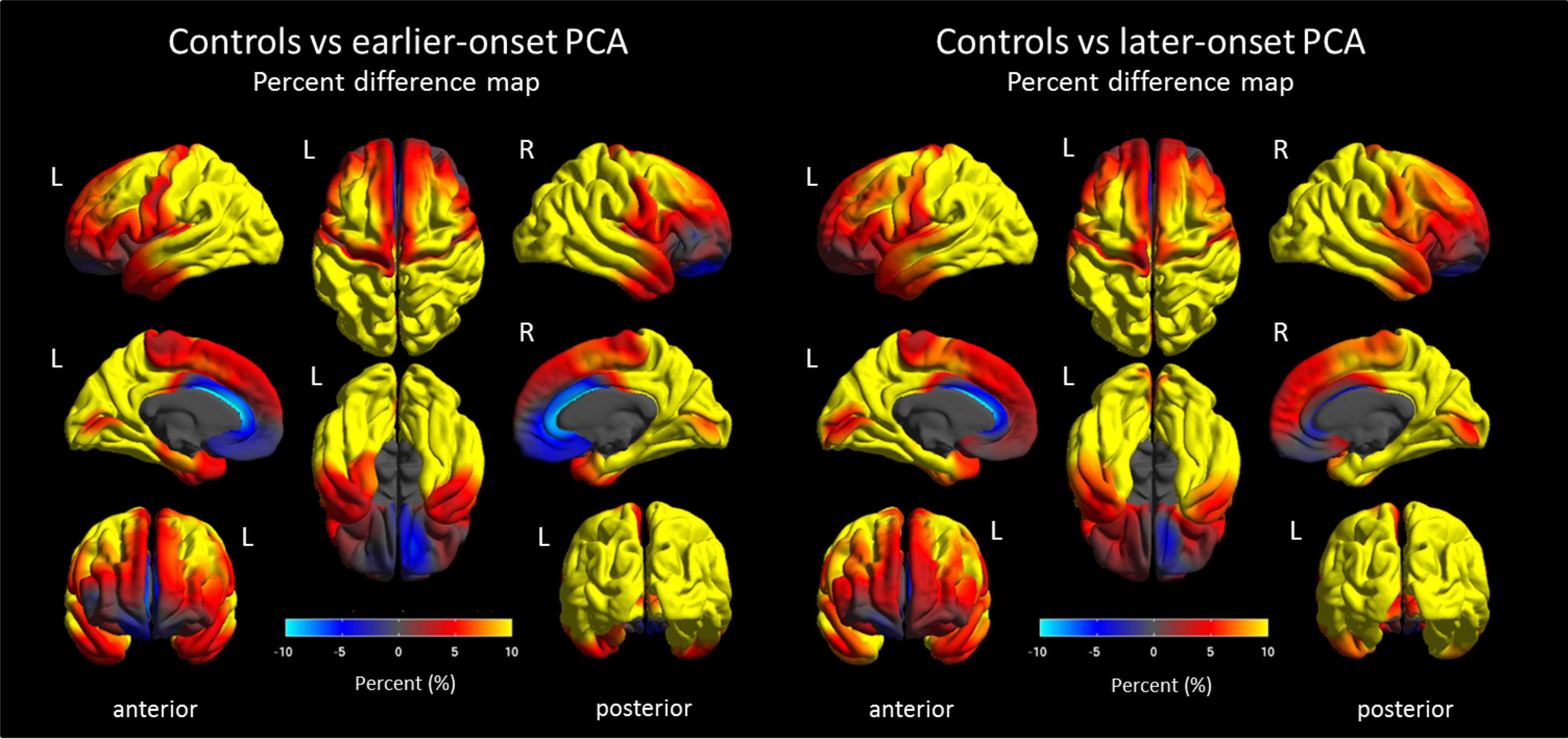

Supplement: Supplementary Figure 1 [file NIHMS68860-supplement-Supplementary_Figure_1.jpg]

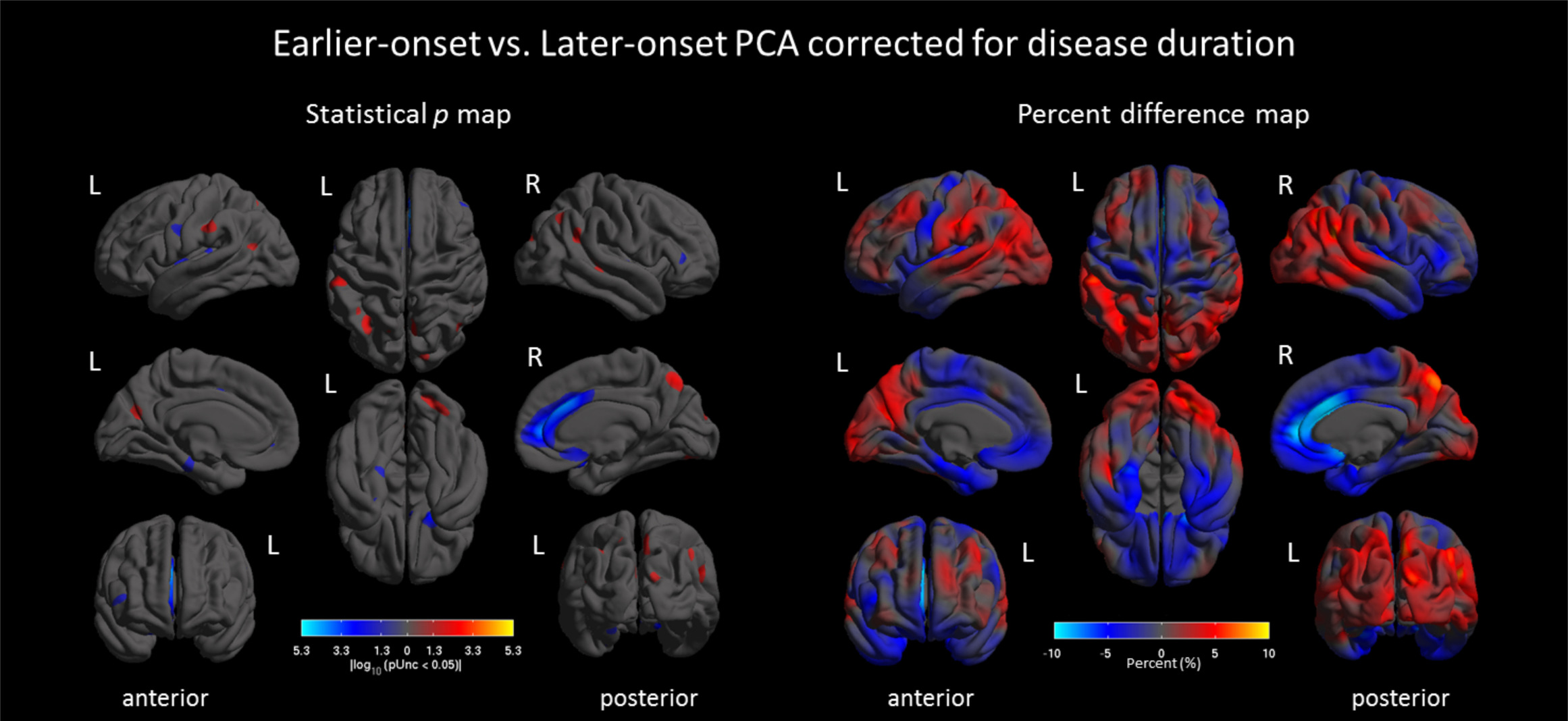

Supplement: Supplementary Figure 2 [file NIHMS68860-supplement-Supplementary_Figure_2.jpg]

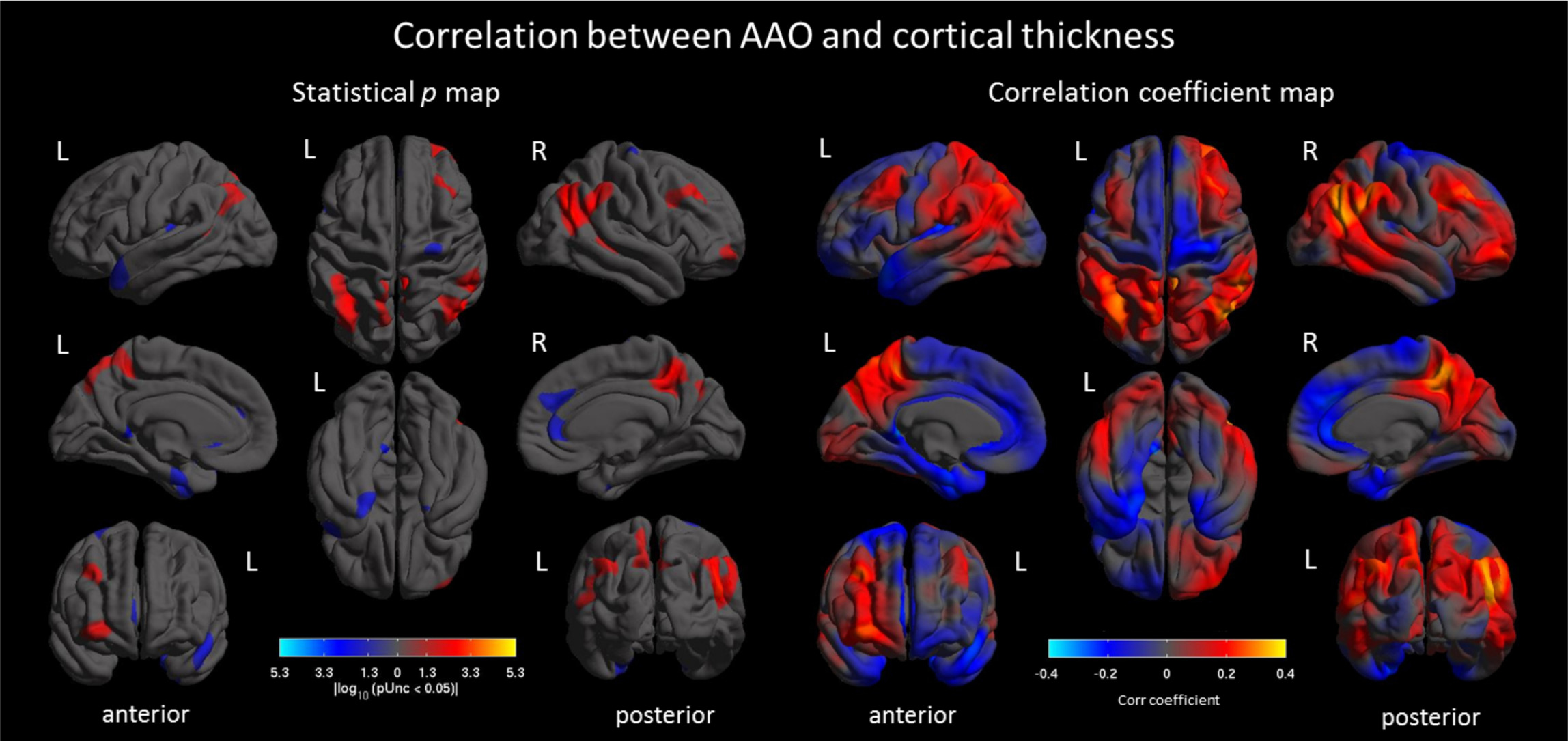

Supplement: Supplementary Figure 3 [file NIHMS68860-supplement-Supplementary_Figure_3.jpg]

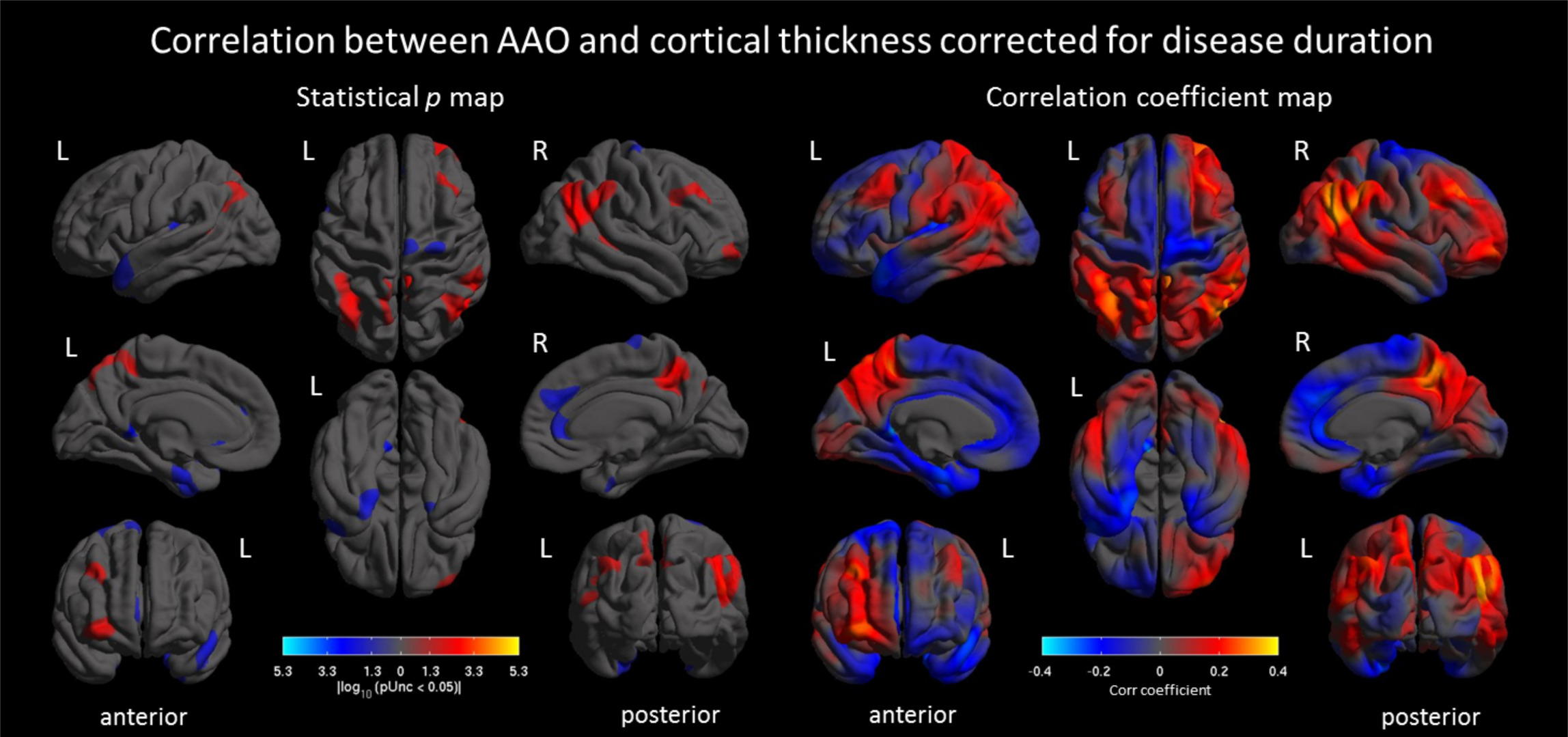

Supplement: Supplementary Figure 4 [file NIHMS68860-supplement-Supplementary_Figure_4.jpg]

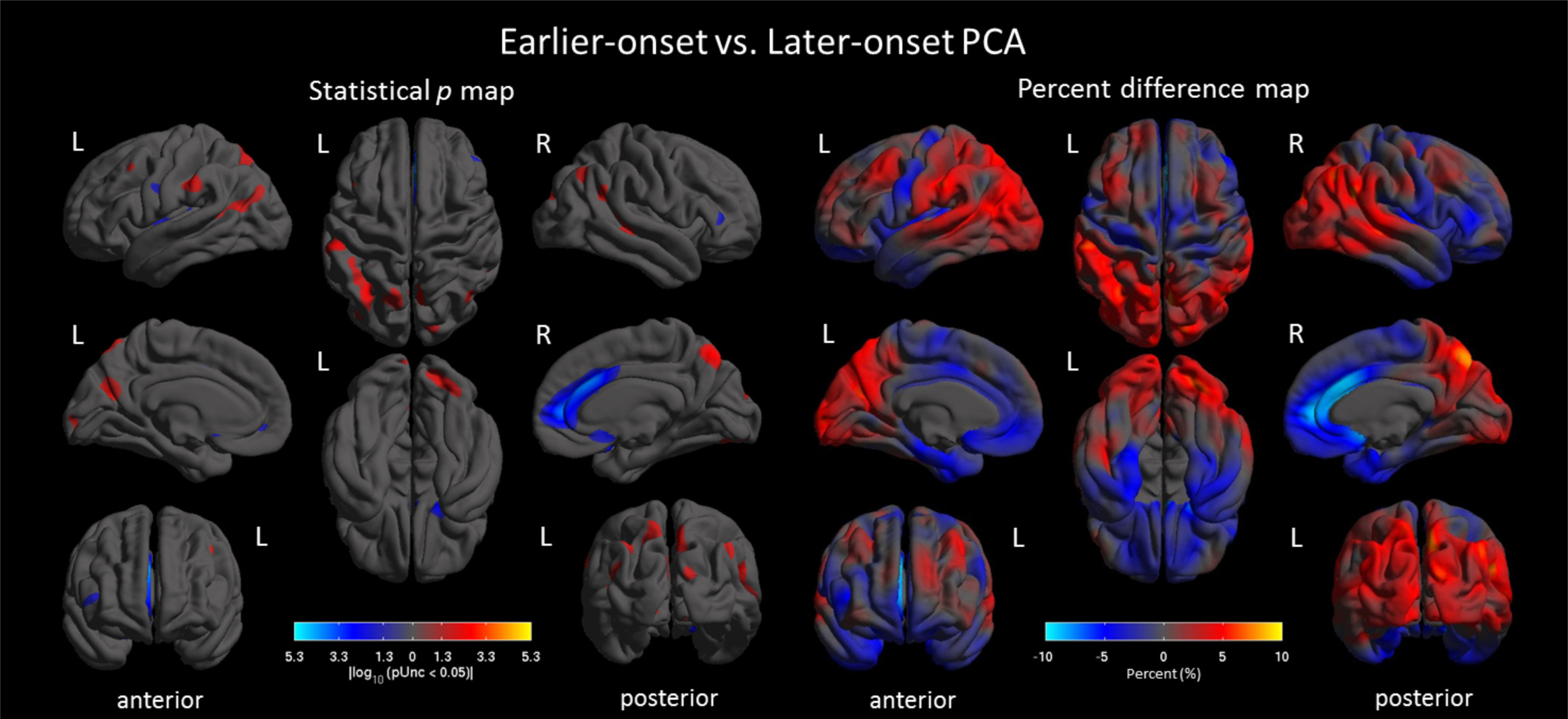

Supplement: Supplementary Figure 5 [file NIHMS68860-supplement-Supplementary_Figure_5.jpg]
