# Supplementary material for: Effect of age at onset on cortical thickness and cognition in posterior cortical atrophy
Source: Neurobiol Aging. Author manuscript; Available in PMC 2016 Aug 1. (PMC4926954; doi:10.1016/j.neurobiolaging.2016.04.012)
Supplement: Supplementary Methods [file NIHMS68860-supplement-Supplementary_Methods.docx]

**Imaging parameters**

T1-weighted volumetric MR scans were acquired on five different scanners at 3 sites:

**Site 1 - DRC:**

a) 1.5T scanners: Scans from two 1.5T scanners were included in this study, both of which used an inversion recovery (IR)-prepared SPGR with a 256x256 image matrix and a field of view of 24cm to provide 124 contiguous 1.5mm coronal slices. Acquisition parameter for scanner #1: TE=6.3ms, TR=14.2ms, TI=650ms, flip angle=15°; parameters for scanner #2: TE=5.2ms, TR=12ms, TI=650ms, flip angle=13°.

b) 3T scanner: Siemens Trio TIM 3T scanner which used an MPRAGE sequence with a 256x256 acquisition matrix and 28.2-cm field of view to provide 208 contiguous 1.1mm slices in the sagittal plane; acquisition parameters: TE=2.9ms, inversion interval TR=2200ms, TI=900ms.

**Site 2 - UCSF:**

Siemens Trio TIM 3T scanner which used an MPRAGE sequence; acquisition parameters: TE=2.98 ms, TR=2300ms, TI=900ms, flip angle=15°, slice thickness 1.5mm.

**Site 3 - HUVR:**

1.5T Philips Intera scanner which used a TFE T1 3D sequence with 288 x 288 acquisition matrix; acquisition parameters: TE= 4ms, TR=8.69ms, flip angle=8°, slice thickness 1 mm
