# Supplementary material for: Effect of age at onset on cortical thickness and cognition in posterior cortical atrophy
Source: Neurobiol Aging. Author manuscript; Available in PMC 2016 Aug 1. (PMC4926954; doi:10.1016/j.neurobiolaging.2016.04.012)
Supplement: Supplementary Table 1 [file NIHMS68860-supplement-Supplementary_Table_1.docx]

| **Supplementary Table 1**. Site and scanner distribution in earlier and later-onset PCA | | | |
| --- | --- | --- | --- |
|  | Field Strength | Earlier-onset PCA (N) | Later-onset PCA (N) |
| DRC | *3T* | 23 | 22 |
|  | *1.5T* | 21 | 15 |
| UCSF | *3T* | 4 | 4 |
|  | *1.5T* | 0 | 0 |
| HUVR | *3T* | 0 | 0 |
|  | *1.5T* | 1 | 8 |
|  | | | |
